# Effectiveness of VIA, Pap, and HPV DNA Testing in a Cervical Cancer Screening Program in a Peri-Urban Community in Andhra Pradesh, India

**DOI:** 10.1371/journal.pone.0013711

**Published:** 2010-10-28

**Authors:** Patti E. Gravitt, Proma Paul, Hormuzd A. Katki, Haripriya Vendantham, Gayatri Ramakrishna, Mrudula Sudula, Basany Kalpana, Brigitte M. Ronnett, K. Vijayaraghavan, Keerti V. Shah, for the CATCH Study Team

**Affiliations:** 1 Department of Epidemiology, Johns Hopkins Bloomberg School of Public Health, Baltimore, Maryland, United States of America; 2 Department of Molecular Microbiology and Immunology, Johns Hopkins Bloomberg School of Public Health, Baltimore, Maryland, United States of America; 3 Division of Cancer Epidemiology and Genetics, National Cancer Institute, Bethesda, Maryland, United States of America; 4 SHARE INDIA, Mediciti Institute for Medical Sciences, Ghanpur, India; 5 Center for DNA Fingerprinting and Diagnostics, Hyderabad, India; 6 Departments of Pathology and Gynecology and Obstetrics, The Johns Hopkins University School of Medicine and Hospital, Baltimore, Maryland, United States of America; University of Cape Town, South Africa

## Abstract

**Background:**

While many studies have compared the efficacy of Pap cytology, visual inspection with acetic acid (VIA) and human papillomavirus (HPV) DNA assays for the detection cervical intraepithelial neoplasia and cancer, few have evaluated the program effectiveness.

**Methods and Findings:**

A population-based sample of 5603 women from Medchal Mandal in Andhra Pradesh, India were invited to participate in a study comparing Pap cytology, VIA, and HPV DNA screening for the detection of CIN3+. Participation in primary screening and all subsequent follow-up visits was rigorously tracked. A 20% random sample of all women screened, in addition to all women with a positive screening test result underwent colposcopy with directed biopsy for final diagnosis. Sensitivity, specificity, positive and negative predictive values were adjusted for verification bias. HPV testing had a higher sensitivity (100%) and specificity (90.6%) compared to Pap cytology (sensitivity  =  78.2%; specificity = 86.0%) and VIA (sensitivity = 31.6%; specificity = 87.5%). Since 58% of the sample refused involvement and another 28% refused colposcopy or biopsy, we estimated that potentially 87.6% of the total underlying cases of CIN3 and cancer may have been missed due to program failures.

**Conclusions:**

We conclude that despite our use of available resources, infrastructure, and guidelines for cervical cancer screening implementation in resource limited areas, community participation and non-compliance remain the major obstacles to successful reduction in cervical cancer mortality in this Indian population. HPV DNA testing was both more sensitive and specific than Pap cytology and VIA. The use of a less invasive and more user-friendly primary screening strategy (such as self-collected swabs for HPV DNA testing) may be required to achieve the coverage necessary for effective reduction in cervical cancer mortality.

## Introduction

Broad adoption of effective Pap smear screening programs is credited with a substantial reduction in cervical cancer incidence in many countries, but it has not been possible to implement this strategy in the developing world, which bears 80% of the global burden of cervical cancer. The reasons for this failure include a lack of infrastructure, requirement of specialized training, requirement of multiple visits by the woman for follow-up and treatment, the difficulties in implementing quality controls for the procedures, and lack of facilities to provide the needed treatment. Therefore, over the last decade, efforts to reduce the global cervical cancer burden through screening have focused on development and evaluation of alternative screening assays to the Pap smear. Two such assays have been widely promoted: visual inspection of the cervix following acetic acid application (VIA) and molecular tests for the presence of high risk human papillomavirus (HR-HPV) infection [1], [2]. VIA offers important potential advantages because the procedure is simple and results are available immediately and in many instances, cryotherapy treatment can be provided at the same visit. HPV DNA testing offers the advantage of an objective assay for the presence of the viruses which are responsible for cervical cancer.

While both of these assays have been rigorously evaluated in controlled research settings [3], [4], [5], [6], [7], [8], [9], [10], few studies have reported on the effectiveness of these alternative assays for detection of pre-neoplastic disease and cancer when implemented in a typical health care delivery setting using limited outside resources. In order to provide a bridge between well-controlled research studies and programmatic implementation, we addressed the following aims in women age 25 years and older in a population-based study in Medchal Mandal, a peri-urban rural community in the state of Andhra Pradesh, India: (1) evaluate the effectiveness of using VIA, PAP, and HPV DNA testing to detect CIN2+ and (2) evaluate the implementation of cervical cancer screening within the local health care system. We used only readily available local resources for training and implementation. The results of this study provide estimates of screening coverage, rates of follow-up visits, performance characteristics of the screening assays, and estimates of the disease burden in the population in a rural region in India.

## Materials and Methods

### Ethical Considerations

The study protocol was approved by the Institutional Review Boards at SHARE India, MediCiti Institute for Medical Sciences and Johns Hopkins University. Written informed consent was obtained by signature or thumbprint.

### Study Setting

REACH, or Rural Effective Affordable Comprehensive Healthcare, was designed by SHARE India, a non-governmental organization, to develop a replicable working model of heath care delivery that offers preventive, primary, and secondary health care to a rural population (http://www.sharehealth.net/). The REACH project is centered in MediCiti Institute of Medical Sciences (MIMS) centrally located in the rural community it serves, 32.4 km north of Hyderabad. Combined use of ambulatory units staffed with a doctor, nurse and health supervisor and a team of community health volunteers (CHVs) facilitates frequent contact with the population in their communities. The REACH project is supported by intensive information technology (IT), with enumeration of the population in the targeted areas specifically, in Medchal Mandal in Ranga Reddy District, Andhra Pradesh. The census data are acquired by household surveys, and contact information as well as pertinent health indicators for the members of each household are maintained by computerized database. These data are updated annually. The combined resource of up-to-date census information and mobile health units afford a unique opportunity for evaluation of screening programs.

### Study design

Using a census list of the Medchal Mandal community, we approached all eligible women from 42 villages to participate in the CATCH Study from January 2005 to July 2007. The total population in the 42 villages was approximately 45,800, with individual village population ranging from 46 to 4712. The villages were located 0.5 to 25 kilometers from the MediCiti Hospital. Individual house-to-house recruitment with personal invitation was conducted in 35 villages; village level invitation was used in the remaining 7 villages. Women were eligible if they were 25 years or older, had an intact uterus, were mentally competent, and were able and willing to provide informed consent.

All consenting women were transported to MediCiti Hospital where they received three tests for early detection of cervical cancer and neoplasia (VIA, Pap smear, and HPV DNA) and were then transported back to their villages. To obtain data for correction of verification bias, 20% of the enrolling women were randomized to receive colposcopy (immediate colposcopy arm) on the day of the enrollment screening exam regardless of screening test results. As soon as the results of all three tests were available, in about 3–4 weeks after the screening visit, the women were contacted at their homes and informed of their test results by a health supervisor. Women who were positive by any one or more of the screening tests (excluding women who were already colposcoped at their first visit) were asked to return to the hospital for a colposcopic examination. The screening and any required treatment were provided at no cost to the participant, who was also provided lunch and 2 kg of rice as an incentive for participation.

### Recruitment

Recruitment was conducted in two phases, systematically covering one village at a time. In the first preparatory phase, the elected village leader (*Sarpanch*) and the MediCiti CHV were contacted by the REACH project community liaison and the project health counselors to explain the project and obtain support to recruit in the village. Preparatory mass education programs on gynecologic health and cervical cancer prevention were delivered to the community prior to systematic house-to-house recruitment efforts. In the second phase, age-eligible women were recruited from their house by a team of health supervisors and health counselors. Additional education and detailed explanation of the screening goals and procedures was provided at that time. This ‘motivation’ phase was conducted in the evenings, and women who expressed interest and willingness to be screened were scheduled to be picked up by hospital vehicles the following morning for transportation to the screening clinic. Women came to the hospital in groups of 5-30 women per day (average 10 per day).

### Screening visit

Upon arrival at the clinic, a printed consent form was read aloud to them as a group, and each woman was privately queried as to her understanding of the consent and given an opportunity to ask questions. Women who agreed to participate provided a signature or thumbprint on the printed consent form in the presence of a witness. After consent, women responded to a brief interviewer-administered questionnaire designed to assess demographic information as well as cervical cancer screening, reproductive, contraceptive, and tobacco use histories.

### Screening test methods

During a speculum examination, trained gynecologists collected (in order) ecto- and endo-cervical cells for Pap smear, exfoliated cervical cells for HPV DNA testing, and evaluated the cervix after acetic acid application (VIA). Any abnormalities found after examination of the vulva, vagina, and cervix (e.g., discharge, inflammation, clinical diagnosis of STI, etc.) were recorded on a standardized pelvic exam form. This form also allowed systematic recording of any prescribed medication as a result of the pelvic exam (e.g., antibiotic, antifungal). For the women randomized to receive colposcopy at enrollment, the colposcopic exam was performed after collection of specimens for Pap smear and HPV DNA testing and after conducting VIA.

### Pap smear collection and interpretation

After removing any obscuring mucus from the cervix with a cotton swab, exfoliated ectocervical cells were collected and smeared onto a glass slide using an Ayres spatula. Endocervical cells were collected by endocervical brush and placed onto the same slide. Cells were fixed by placing the slides in ethanol. Slides were stained according to standard protocols, and reviewed by a trained local cytopathologist, who recorded the cytologic diagnosis on standardized forms according to the 2001 Bethesda System [11]. Women with a cytologic diagnosis of ASC-US or more severe lesion were scored as Pap smear positive. We considered the few women with unsatisfactory Pap smear results as Pap negative (n = 86; 3.8%) rather than recalling them for a repeat Pap smear, since logistically repeating the Pap in resource poor areas will not be feasible.

### HPV sample collection, detection, and interpretation

Following collection of the Pap smear, a Digene conical brush sampler was placed in the cervical os, rotated 360° three times, removed, and placed into 1 ml of Digene standard transport medium (STM). Samples were stored at 4°C for no more than 24 hours after collection before aliquoting and long term storage at −20°C. HPV DNA testing was performed locally using the hybrid capture 2 (hc2) test according to the manufacturer's instructions. Samples with an RLU/CO value ≥1.0 were considered to be positive for high risk HPV.

### VIA methods and interpretation

Using standard interpretation guides [12], a positive VIA outcome was defined as “sharp, distinct, well-defined, dense (opaque, dull, or, oyster white) aceto-white areas with or without raised margins, abutting the squamo-columnar junction in the transformation zone” or “strikingly dense aceto-white areas in the columnar epithelium” or “condyloma and leukoplakia occurring close to the squamo-columnar junction turning intensely white” 1 minute after the application of a 5% acetic acid solution.

### Follow-up and colposcopy

Women requiring colposcopic examination because of a positive screening test result were contacted at their homes, and a hospital vehicle was provided for transportation to and from the clinic. The colposcopist was aware that referred patients had at least one positive screening test, but was masked to the specific VIA, Pap, and HPV DNA results. Even though the VIA test results were available immediately at the time of screening, referral to colposcopy was made only after all three test results were available. Biopsies were taken from any suspicious lesion. All biopsies were read locally, and were subsequently reviewed by an expert pathologist at Johns Hopkins Medical Institutions (JHMI). Women with histologically confirmed cervical intraepithelial neoplasia (CIN) grade 2 or 3 by either local or JHMI review were referred for treatment by LEEP/cold knife conization or hysterectomy. Women found to have operable invasive cancer were treated at MIMS. Women requiring radiotherapy were referred to the government cancer hospital. A release form was signed by any woman with CIN2+ who refused treatment.

### Statistical Methods

Differences in test positivity by age were assessed using Pearson's chi-square tests. Test agreement was measured using kappa statistics with 95% confidence intervals. The agreement between the JHMI and MIMS diagnosis of <CIN2 vs. CIN2+ was good (94.9% total agreement, 60.9% percent positive agreement). For this analysis, cases were defined based on the JHMI pathology diagnosis. Diagnostic accuracy statistics (sensitivity, specificity, and positive/negative predictive values (PPV/NPV) were calculated for each of Pap, VIA, and HPV testing for each of CIN2+ and CIN3+ outcomes. Crude estimates of diagnostic accuracy were biased due to non-random exclusion of women who did not have the opportunity for full diagnostic verification (i.e., women who screened negative and were not randomized, or, referred women who refused colposcopy and/or biopsy) [13]. To properly account for this verification bias, we used inverse-probability weighting to weight up women with observed histology to represent the full cohort of 2331 women. We extended methods previously developed to account for verification bias under stratified two-phase sampling to three-phase sampling with sampling strata defined by the eight combinations of Pap, VIA, and HPV test results (e.g. +++, ++−, etc.). First, the 670 women who accepted a biopsy and had histology results were weighted up (within sampling strata) to represent all 781 women who appeared for colposcopy. Second, the 781 women who appeared for colposcopy were weighted up to represent the full cohort of 2331 women who consented. This correction for verification bias assumes that women who refused a colposcopic exam or who refused an indicated biopsy have the same disease rates as women who accepted the exam and biopsy, respectively, within each sampling stratum.

## Results

### Participation in screening and diagnostic follow-up procedures

**Figure 1:**
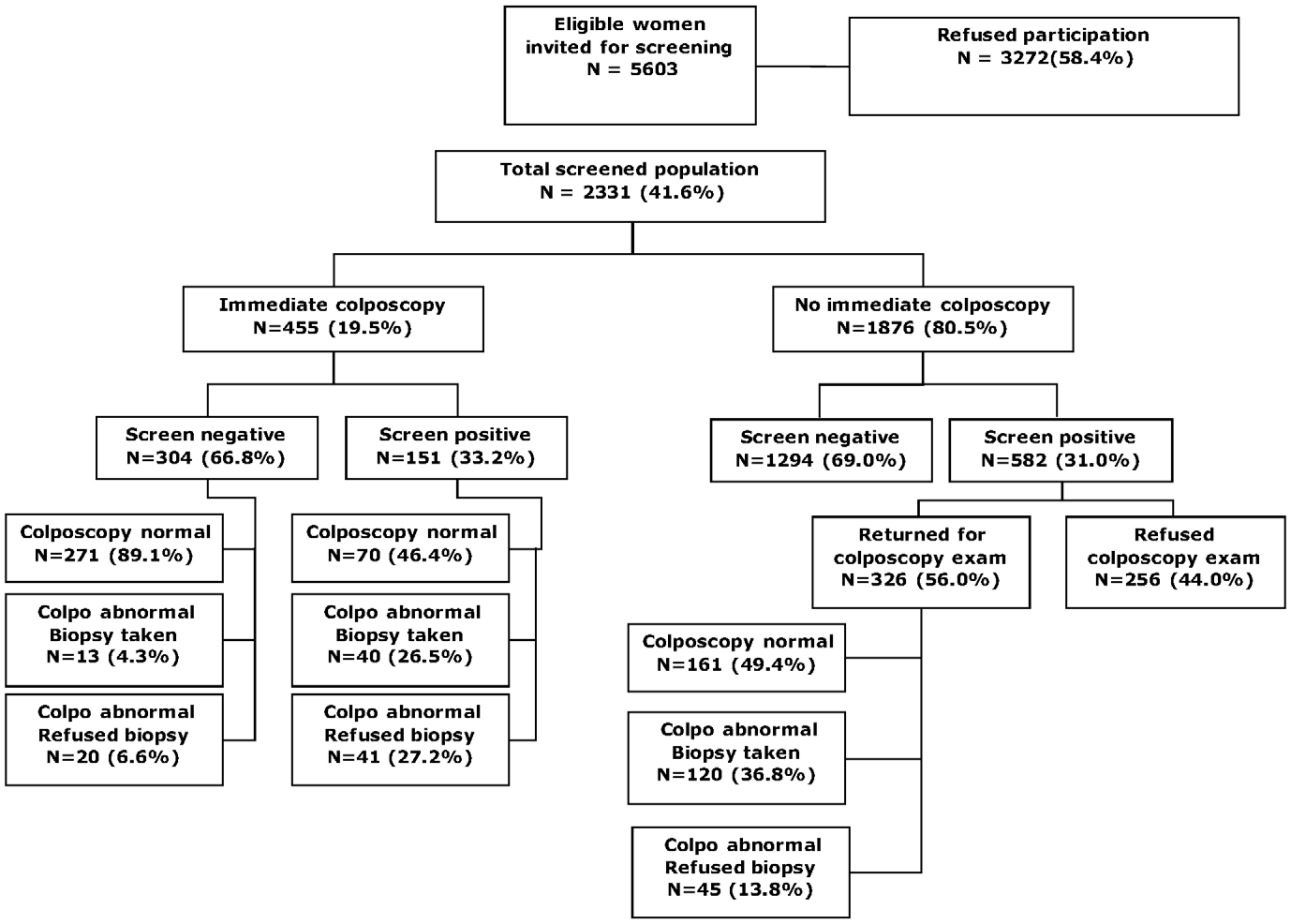
Participation in screening and follow-up (colposcopy and biopsy where recommended) by randomization arm and screening result. Screen positive indicates positive result on VIA, Pap, and/or HPV DNA testing. Colposcopy normal indicates no area of abnormality identified, no biopsy recommended. Colposcopy abnormal-biopsy taken indicates that a biopsy was successfully obtained from all visually identified areas of abnormality. Colposcopically abnormal-biopsy refused indicates that a lesion was visualized and biopsy recommended, but the patient refused.

The degree of participation in the study and compliance with screening and diagnostic follow-up is summarized in Figure 1, stratified by immediate colposcopy arm and by overall screening test results. A total of 5603 women were determined to be eligible for screening and were invited to participate by at least one person-to-person contact. Of these, 2331 (41.6%) enrolled and completed the screening protocol; the remainder refused participation. As targeted in the study protocol, approximately 20% of consenting women were randomized to receive an immediate colposcopic examination following their screening tests at the enrollment visit (455/2331, immediate colposcopy arm). In addition, 582 women not randomized to immediate colposcopy but testing positive by any one or more of the three screening tests were referred back for a colposcopic examination at a second visit. Disease ascertainment was incomplete in both arms. In the randomized arm, a total of 114 women (25%) had a colposcopic abnormality where a biopsy was indicated; of these women, 61 (53.5%) refused biopsy. In the referred arm, 582 of the 1876 women (31%) were screen-positive and were referred for colposcopy; of these, 256 women (44.0%) did not return for the colposcopic follow-up. Among those who did come for the follow-up visit, 165 (50.6%) had a colposcopic abnormality indicating biopsy, with 45 (27.3%) refusing biopsy (significantly lower than biopsy refusal among women with immediate colposcopy (53.4%, p<0.001).

### Baseline demographics

The baseline demographics of the enrolled population are presented in Table 1. Consistent with the age-specific participation rates, the enrolled cohort was skewed toward younger ages, with 48% of women between 25–34 years (mean age 37.4 years, SD 11.1). Most participants identified themselves as Hindu (86.8%) and had no formal education (69.4%). Most women were either unemployed/housewives (32.6%) or worked in agriculture (39.3%). All women were married (either at the time of enrollment or in the past); therefore we considered the age women first lived with husband (*shobhanam)* as the best surrogate marker of sexual debut. These data were normally distributed with a mean age at shobhanam of 15.6 years (SD 2.8). A few women (13.1%) reported use of tobacco products, and the majority of this was pan (betel leaf) use (93.4%). A more substantial fraction reported passive tobacco smoke exposure (37.9%). Most women were parous (96.8%), with a median of 3 live births. Most women (95.5%) reported no previous pap screening.

**Table 1:** Demographics of study population.

	N	%		N	%
**Age (years)**			**Use of any tobacco (self)**		
25–29	659	28.3%	No	2026	86.9%
30–34	460	19.7%	Yes	305	13.1%
35–39	351	15.1%	**Live with a smoker**		
40–44	256	11.0%	No	1448	62.1%
45–49	199	8.5%	Yes	883	37.9%
50–54	131	5.6%	**Parity**		
55–59	106	4.5%	0	75	3.2%
60+	133	5.7%	1	128	5.5%
missing	36	1.5%	2	569	24.4%
**Religion**			3–4	1094	46.9%
Hindu	2023	86.8%	5+	465	19.9%
Muslim	99	4.2%	**Previous Pap history**		
Christian	208	8.9%	None	2223	95.4%
Other	1	0.0%	Yes	27	1.2%
**Education (highest level completed)**			don't know	79	3.4%
none	1606	68.9%	missing	2	0.1%
1–8	435	18.7%	**Age first lived with husband (years)**		
9 or more	272	11.7%	<10	12	0.5%
missing	18	0.8%	10–13	589	25.3%
**Occupation (self)**			14–16	897	38.5%
housewife/unemployed	702	30.1%	17–19	570	24.5%
laborer	229	9.8%	20–36	213	9.1%
agriculture	846	36.3%	missing	50	2.1%
self-employed	163	7.0%			
government employee	18	0.8%			
private company/other	193	8.3%			
missing	180	7.7%			

### Test positivity by age

The population prevalence of positive results was similar by screening method (12.7%, 14.6%, and 10.3% for VIA, Pap, and HPV, respectively). HPV prevalence did not vary significantly by age (p = 0.44), while Pap prevalence increased significantly with increasing age (p<0.001)) (Table 2). VIA prevalence did not vary by age among women 25–60 years (p = 0.59), but was significantly higher among women over age 60. We recognize that once- or twice-in-a-lifetime screening is usually recommended for women in a more narrow age range (e.g., 25–50 years). When we restricted our analysis to women in that age range, the prevalence of positive test results was more similar across testing methods (12.1%, 11.1%, and 10.0% for VIA, Pap, and HPV, respectively).

**Table 2:** Age-specific positive screening test prevalence.

	TOTAL	VIA positive	Pap positive	HPV positive
AGE (years)	*N*	*%*	*%*	*%*
25–29	659	10.2%	7.9%	10.5%
30–34	460	13.7%	9.1%	10.9%
35–39	351	12.8%	10.3%	8.0%
40–44	256	11.7%	11.7%	9.0%
45–49	199	14.1%	25.1%	11.6%
50–54	131	13.7%	32.1%	15.3%
55–59	106	12.3%	34.9%	8.5%
60+	133	24.8%	36.8%	10.5%
TOTAL	2295	12.9%	14.7%	10.3%

These estimates exclude 36 women with missing age.

### Concordance of test positive

A total of 733 (31.4%) women were positive by one or more tests, while only 16 (0.7%) were positive for all three tests. The agreement was better between Pap and HPV DNA tests compared to either of these assays and VIA, but agreement beyond that expected by chance was poor in all comparisons (kappa range 0.04–0.11). In order to better understand the disagreement between test results, we tested approximately 19% of VIA- and Pap-positive, but hc2 negative samples using consensus primer PCR [14], [15]. Only 2 of 52 (4%) VIA positive/hc2 negative samples, but 19 0f 95 (20%) of Pap positive/hc2 negative samples tested positive for HPV by PCR, mostly for low-risk HPV types.

### Crude test performance for detection of CIN2/3/cancer

We detected a total of 19 CIN2+ cases; 8 CIN2, 7 CIN3, and 4 invasive cancers. We first calculated the crude assay performance only among those women who had a colposcopic examination and did not refuse biopsy when indicated (N = 675); normal colposcopy results were considered to be negative for CIN2+. Our results show clear performance differences between the assays (Table 3A), with HPV DNA testing having the best sensitivity and specificity when defining cases as CIN2+ or CIN3+ (84.2% and 81.3% for CIN2+; 100% and 80.72% for CIN3+). The next best test was Pap with lower sensitivity (63.2% and 81.8% for CIN2+ and CIN3+, respectively) and specificity (76.2% and 76.1% for CIN2+ and CIN3+, respectively) compared to the HPV DNA test. VIA demonstrated both poor sensitivity and specificity in this analysis (26.3% and 76.4% for CIN2+ and 36.4% and 76.5% for CIN3+). Accordingly, the HPV DNA test showed both high positive predictive value (11.5%) and negative predictive value (99.4%) for CIN2+.

**Table 3:** Test performance characteristics.

A. Among women with full colposcopy and histologic evaluation (N = 675)									
		Sensitivity	(95% CI)	Specificity	(95% CI)	PPV	(95% CI)	NPV	(95% CI)
CIN2+	Pap	63.20%	(38.4%–83.7%)	76.20%	(72.8%–79.4%)	7.14%	(3.75%–12.1%)	98.60%	(97.2%–99.4%)
	VIA	26.30%	(9.2%–51.2%)	76.40%	(72.9%–79.6%)	3.13%	(1.02%–7.14%)	97.30%	(95.5%–98.5%)
	HPV	84.20%	(60.4%–96.6%)	81.30%	(78%–84.2%)	11.50%	(6.72%–18%)	99.40%	(98.4%–99.9%)
CIN3+	Pap	81.82%	(48.22–98.72%)	76.05%	(72.62–79.25%)	5.36%	(2.48–9.93%)	99.61%	(98.58–99.95%)
	VIA	36.36%	(10.93%–69.21%)	76.51%	(73.09%–79.68%)	2.50%	(0.69%–6.28%)	98.64%	(97.22%–99.45%)
	HPV	100.00%	(71.51%–100.00%)	80.72%	(77.51%–83.66%)	7.91%	(4.02%–13.72%)	100.00%	(99.31%–100.00%)

B. Among total screened population, verification bias adjusted estimates (N = 2331)									
		Sensitivity	(95% CI)	Specificity	(95% CI)	PPV	(95% CI)	NPV	(95% CI)
CIN2+	Pap	46.50%	(28.67%–65.30%)	86.04%	(85.59%–86.47%)	7.72%	(5.04%–11.66%)	98.46%	(97.08%–99.20%)
	VIA	16.65%	(8.53%–29.98%)	87.36%	(87.10%–87.61%)	3.20%	(1.74%–5.83%)	97.66%	(96.30%–98.53%)
	HPV	61.21%	(38.45%–79.95%)	90.98%	(90.46%–91.48%)	14.56%	(10.07%–20.60%)	98.94%	(97.52%–99.55%)
CIN3+	Pap	78.24%	(53.48%–91.84%)	85.88%	(85.55%–86.20%)	5.24%	(3.22%–8.42%)	99.75%	(99.29%–99.91%)
	VIA	31.56%	(16.32%–52.16%)	87.45%	(87.26%–87.63%)	2.45%	(1.28%–4.65%)	99.23%	(98.64%–99.56%)
	HPV	100.00%	(na)	90.60%	(90.20%–90.99%)	9.60%	(6.14%–14.72%)	100.00%	(na)

A. Crude estimates among women with full colposcopic and histologic evaluation (N = 675). B. Verification-biased adjusted estimates among total screened population (N = 2331).

CIN: cervical intraepithelial neoplasia, CI: confidence interval, PPV: positive predictive value, NPV: negative predictive value

*Adjusted test performance.* Using inverse-probability weighting, we applied the CIN2+ and CIN3+ rates from women with colposcopy-biopsy results to the full cohort, as described in Methods. One case of CIN2 was detected among the 304 screen-negative women in the immediate colposcopy arm, for a disease prevalence of 0.33%. The verification bias adjusted estimates of test performance are shown in Table 3B. The relative performance of HPV>Pap>VIA was similar to that observed in the crude estimate, though as expected the sensitivity estimates for each test decreased while specificity estimates increased, especially when cases included CIN2 lesions. HPV testing remained more sensitive and specific (sens = 61.2% and spec = 90.9%), compared to VIA (sens = 16.7%; spec = 87.4%) and Pap (sens = 46.5%; spec = 86.0%). Sensitivity was increased substantially for all three tests when CIN2 was removed from the case definition (100.0%, 78.2%, and 31.6% for HPV, Pap, and VIA, respectively).

### Estimates of disease burden in the population

**Figure 2:**
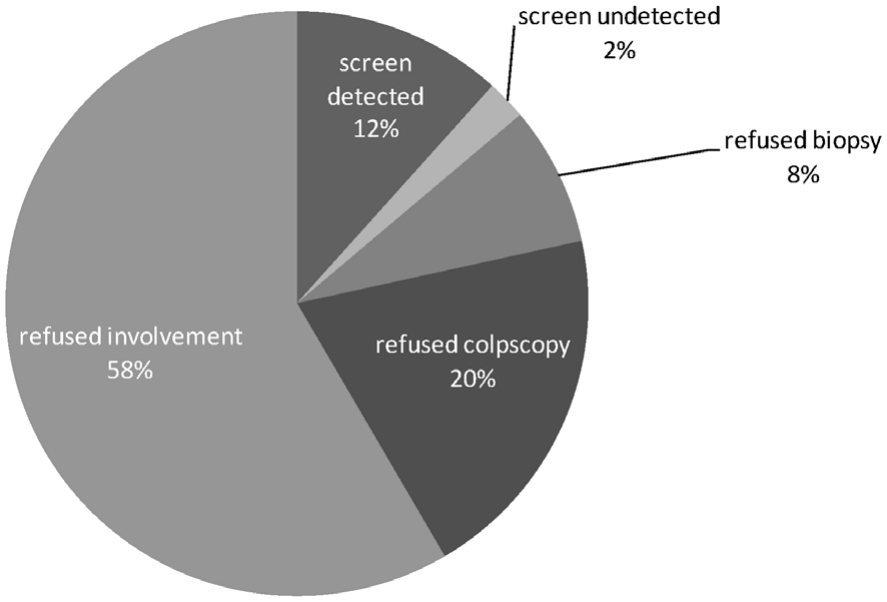
Estimated proportion of cases of CIN2+ observed and estimated via population weighting for verification bias adjustment. *Screen detected* indicates proportion of cases of CIN2+ detected by hc2, and *screen undetected* indicates the proportion of CIN2+ cases detected through the screening program, but missed by hc2. *Refused biopsy* indicates the proportion of CIN2+ cases estimated among those who refused biopsy, *refused colposcopy* indicates the proportion of CIN2+ cases estimated among those who screened positive, but refused colposcopic exam, and *refused involvement* indicates the proportion of CIN2+ cases estimated among those who refused participation in the program (i.e., not screened).

Using the data generated from applying the probability weights to the entire eligible cohort (N = 5603), we estimated a CIN2+ prevalence of 3.8% and a CIN3+ prevalence of 1.6%. This model predicts that 57.1 ‘true positive’ cases of CIN2+ (23 cases of CIN3+) would have been detected with complete follow-up (100% colposcopy and 100% biopsy where indicated), and an additional 137.3 ‘true positive’ cases of CIN2+ (55.4 cases of CIN3+) would have been detected if the entire eligible population had been screened. Figure 2 summarizes the estimated burden of CIN2+ in the population in relation to the cases detected by the hc2 assay.

### Treatment

Of the 19 women who had CIN2+, we provided LEEP for 2, hysterectomy for 9, and referral to the cancer hospital for radiation therapy for 4 women who had invasive cancer. Four women refused treatment despite several direct visits by the study gynecologist for direct counseling.

## Discussion

When evaluated as a complete program, the greatest threat to realizing a reduction in cervical cancer mortality was non-participation in the program and among those who participated, non-compliance with some aspects of the screening requirements, rather than the use of a less accurate screening test. Using standard population-weighted methods to correct for incomplete verification of disease status, we estimated that 86.2% of the potential underlying cases of CIN2+ may have been missed due to program failures; 27.8% as a result of incomplete compliance with follow-up procedures (e.g., colposcopy and biopsy), and 58.4% as a result of non-participation in the screening program. We therefore conclude that despite our use of available resources, infrastructure, and guidelines for cervical cancer screening implementation in resource limited areas developed by the Alliance for Cervical Cancer Prevention (ACCP) [12], community participation and non-compliance remain the major obstacles to successful reduction in cervical cancer mortality in this Indian population and that a more user-friendly screening strategy which reduces the need for a clinic visit may vastly increase coverage.

Participation even in the primary screening phase in our study was low (38%). Evaluations of the screening test alternatives reporting coverage typically show participation rates at or above the 70% threshold thought to be required for effective reductions in cervical cancer mortality [9], [10], [16], [17], [18], [19]. One exception is the study by Nene, et al which reported participation of 56.4% [20]. We cannot readily explain the lower participation in our study compared to other reports of cervical cancer screening in India. One possibility is publication bias, where only studies with participation nearing the 70% effectiveness threshold report participation. In our review of 21 studies comparing alternative screening methods, the majority (14/21, 67%) did not report participation rates at all [5], [6], [8], [9], [10], [16], [17], [18], [20], [21], [22], [23], [24], [25], [26], [27], [28], [29], [30], [31], [32].

A detailed assessment of reasons for refusal will be described separately; in general, older women and women with lower household income were least likely to participate. We conducted focus groups to understand the attitudes of the women in our population toward participation in the screening program. Reluctance to participate was related to the perception that there was no need to go the clinic when they had no symptoms. When probed further, they cited fear/anxiety as a significant factor for reluctance to participate; fear of a cancer diagnosis, of pelvic examination and of community gossip and perception (unpublished data). We revised our educational material in response to the concerns elicited through the focus group. While we did not see any change in participation among women with the lowest reported income, participation improved in the middle- and high-income groups as a result of this intervention.

The reasons for non-participation were very similar to those reported by Basu [33] in cervical cancer screening in Kolkata. Specifically, in the Kolkata study, among women declining screening by their own choice, 46.1% cited ‘I do not need any check up since I have no complaint’ as the reason for not attending the screening visit. Other commonly cited reasons for non-participation in their standardized survey included fear of the tests (36.2%), adverse effects of the screening procedure reported by neighbor/relative (27.6%), feeling shy to have the exam (26.7%), and a desire to let fate/God guide destiny (18.5%) [33]. It was interesting to note that some women who declined to participate in the Calcutta study stated that they would ‘get the test done in private institutions with better facilities’. It is possible that in communities with access to multiple health care venues, as is the case in our study population in Medchal Mandal (including private hospitals, NGO-affiliated hospitals, and government hospitals), competition and perceived quality of care could be influential in choice of cervical cancer screening programs.

Among women screened, substantial differences in test performance for Pap smear, VIA, and HPV DNA tests were observed. As expected, adjustment for verification bias decreased sensitivity and increased specificity for all tests. The sensitivity estimates for HPV testing, particularly for detection of CIN3+, are generally consistent with prior studies [4], [7], [8], [9], [34], [35], while the sensitivity for VIA was remarkably lower than previous reports. The lower sensitivity of VIA was explored in detail in a separate manuscript [36] which showed a strong inter-rater variability as well as a non-specific reactivity in the presence of inflammation. A second possible explanation for our lower sensitivity estimates of VIA could be related to study design. Specifically, because we did not use a screen-and-treat approach in the VIA arm, referral of women testing positive by VIA was delayed by the same interval as those testing positive by hc2 and Pap cytology. This minimized any bias that resulted from lesions that regressed between screening and follow-up [37] and the correlative bias between VIA and colposcopy which rely on similar visual clues [13], [38], which could have led to an overestimation of sensitivity in other studies. Alternatively, VIA is not recommended for women over age 50 years. We did not place an upper age restriction for participation in this study because we felt that in our population of women who had never been screened, everyone could benefit from at least one of the 3 screening tests (HPV testing performance is not compromised in older women). Since 6/19 (31.6%) of our cases would have been lost with age restriction, we presented the unrestricted performance data. However, inclusion of women over age 50 years could have led to an underestimated sensitivity for VIA. The sensitivity of Pap testing at an > =  ASC-US threshold was moderate. We note that the specificity of Pap smears was lower than most studies, which is likely attributable to the increase in ASC-US diagnosis in older women (Table 2), possibly the result of misinterpretation of cellular changes secondary to hormonal declines during menopause. We evaluated the use of alternative thresholds for Pap positivity (eg., LSIL, HSIL) to improve the test specificity, but found these to result in a significant reduction in test sensitivity (data not shown). The fact that HPV PCR was positive (predominately with low risk types) in a higher proportion of Pap positive/hc2 negative samples, compared with VIA positive/hc2 negative samples, suggests that while most of the false positive VIA results were unrelated to HPV-associated changes, some positive Pap smears may reflect cellular changes due to infection with low-risk HPVs.

When we removed CIN2 from the case definition, the performance estimates increased for all tests. Verification bias-adjusted sensitivity for CIN3+ was 100.0% for HPV, 78.2% for Pap, and 31.6% for VIA. The uncertainty of CIN2 diagnoses in detecting women with true precancerous lesions is well-described [39]. In our study, two of three hc2-negative CIN2 cases were also negative for 37 HPV genotypes detected by PCR (Roche Linear Array) in swabs collected at both the screening and the diagnostic visits. This suggests that the morphologic changes were unrelated to HPV and were misclassified as CIN2. Such misclassification is magnified in verification bias-adjusted estimates, raising questions as to the validity of test performance metrics when including CIN2 in the case definition. A case definition which combines morphologic and molecular criteria (e.g., p16 staining, multiple HPV tests) may be useful for future evaluations of screening test performance to avoid misclassification bias.

Our study has limitations. The low number of cases detected in our study likely reflects an under-ascertainment of disease. Our case definition was based on histologically confirmed CIN2+ from colposcopically-directed biopsy. This is a flawed reference standard [40] and likely to miss a substantial proportion of underlying CIN2+ lesions [34], [38]. Specifically, we detected 19 cases of CIN2+ in our study, representing an overall prevalence among the population of women with full colposcopic and histologic evaluation of 2.8% (CIN2/3 = 2.2%; cancer = 0.6%). The observed CIN2+ prevalence among all fully screened women was 0.8% (CIN2/3 = 0.6%; cancer = 0.2%). We note, however, that these estimates are largely consistent with those in a large study of over 140,000 women in Maharashtra State which reported CIN2-3 prevalence ranging from 0.7%–1.0% and cancer prevalence ranging from 0.2%–0.3% [19]. In comparison to the observed prevalence, our estimates of prevalence following inverse probability weighting to the entire population were somewhat higher (3.8% and 1.6% for CIN2+ and CIN3+, respectively). These prevalence estimates may be inflated, since we did not further stratify the subgroups by age and other determinants of CIN2+. The small number of observed cases precluded additional levels of stratification for the inverse probability weighting.

Compliance with follow-up colposcopy and biopsy were also low in our study. Losses to follow-up are well-described barriers to cervical cancer screening in resource-limited populations, and therefore the colposcopic refusal results are not entirely unexpected. Our follow-up may have been lower than that reported in other studies [9], [19] because we were unable to provide colposcopic exams at peripheral field sites. However, follow-up rates were similar by village and did not decrease with increasing distance from the hospital. Women were approached up to 3 times to encourage participation in colposcopy, sometimes by the study gynecologist, before we considered the participant lost to follow-up. When we probed for reasons for non-compliance with follow-up in focus groups, the majority reported that they would wait until they developed symptoms. A few suggested that they were seeking treatment outside of the study. We required verbal consent prior to taking biopsies, which may have reduced compliance with this procedure. We did not systematically document reasons for refusing biopsy. The higher biopsy refusal in the group who were randomized to immediate colposcopy could represent a mix of women who would and would not have returned for a follow-up colposcopy if referred.

Our study also has several strengths. Use of the census data from an entire Mandal allowed us to calculate population-based estimates of test performance as well as compliance with screening and diagnosis. In addition, each woman received all three screening tests, allowing for direct comparison of test performance, which was not possible in large community randomized trials. Use of PCR testing to confirm discrepant results also enhanced our understanding of the lack of correlation in the screening test results by providing a second objective measure.

In conclusion, our results suggest that currently proposed algorithms for cervical cancer screening in resource limited regions are still plagued by infrastructural and compliance barriers. We have previously reported that participation rates may increase substantially if the primary screening is offered in the village by self-collection of samples for HPV testing [41]. Programmatic changes such as village-based self-sampling may therefore offer alternatives strategies for increased participation in cervical cancer screening in resource poor regions. However, successful reduction of cervical cancer burden will require development of acceptable strategies for follow-up of screen positive women. For example, in our study[41] only 34.8% of women who were found to be HPV positive as a result of village-based self-sampling returned for colposcopic examination. Finally, establishment of regional programs of cervical cancer screening of older women linked with programs for immunization of younger women may be expected to reduce the burden of cervical cancer in resource-limited regions of the world. The tools to substantially reduce the global burden of cervical cancer are available or will soon become available. Realistic evaluation of sustainable program implementation will be critical to ensure that the tools are used to maximum benefit with the least cost.
